# Ventricular CSF-to-blood water transport kinetics in adult hydrocephalus

**DOI:** 10.1177/0271678X261466792

**Published:** 2026-06-29

**Authors:** Per Kristian Eide, Alexander Gul Sherwani, Luis Romundstad, Jan Gunnar Fjeld, Amanda Eriksson, Trine Hjørnevik, Markus Hovd

**Affiliations:** 1 Department of Neurosurgery, Oslo University Hospital – Rikshospitalet, Oslo, Norway; 2 Faculty of Medicine, Institute of Clinical Medicine, University of Oslo, Oslo, Norway; 3 KG Jebsen Centre for Brain Fluid Research, University of Oslo, Oslo, Norway; 4 Department of Nuclear Medicine, Oslo University Hospital – Rikshospitalet, Oslo, Norway; 5 Division of Emergencies and Critical Care, Department of Anesthesia and Intensive Care Medicine, Oslo University Hospital, Oslo, Norway; 6 Department of Physics and Computational Radiology, Oslo University Hospital, Oslo, Norway; 7 Department of Pharmacy, University of Oslo, Oslo, Norway; 8 Department of Transplantation Medicine, Oslo University Hospital, Oslo, Norway

**Keywords:** Ventricular water absorption, ventricular infusion test, cerebrospinal fluid, intracranial pressure, ^15^O-labelled water

## Abstract

Water exchange between cerebrospinal fluid (CSF) and blood is fundamental to brain fluid homeostasis, yet ventricular CSF-to-blood water transport has never been quantified as a physiological parameter in adult humans. We investigated ventricular water absorption kinetics in 40 adults undergoing diagnostic evaluation for hydrocephalus. During ventricular infusion testing, ^15^O-labelled water was administered intraventricularly via an external ventricular drain, with continuous jugular bulb blood sampling and simultaneous intracranial pressure (ICP) monitoring. Ventricular water absorption was estimated using non-parametric population pharmacokinetic modelling of venous radioactivity–time curves. Model performance demonstrated robust individual parameter estimation. Ventricular water absorption showed substantial inter-individual variability (mean half-time 65.2 ± 34.3 s; range 31.5–196.8 s). Absorption kinetics were not associated with static or pulsatile ICP metrics (overnight or during infusion), ventricular volume or surface area, or CSF–blood osmotic gradients. No associations were observed with demographic variables. A modest positive association was detected with the CSF–blood protein concentration difference. These findings establish ventricular CSF-to-blood water transport as a quantifiable physiological metric in adult hydrocephalus and suggest that ventricular water exchange is regulated independently of global intracranial pressure and ventricular morphology, consistent with intrinsic barrier and microvascular exchange mechanisms.

## Graphical abstract

**Graphical Abstract:**
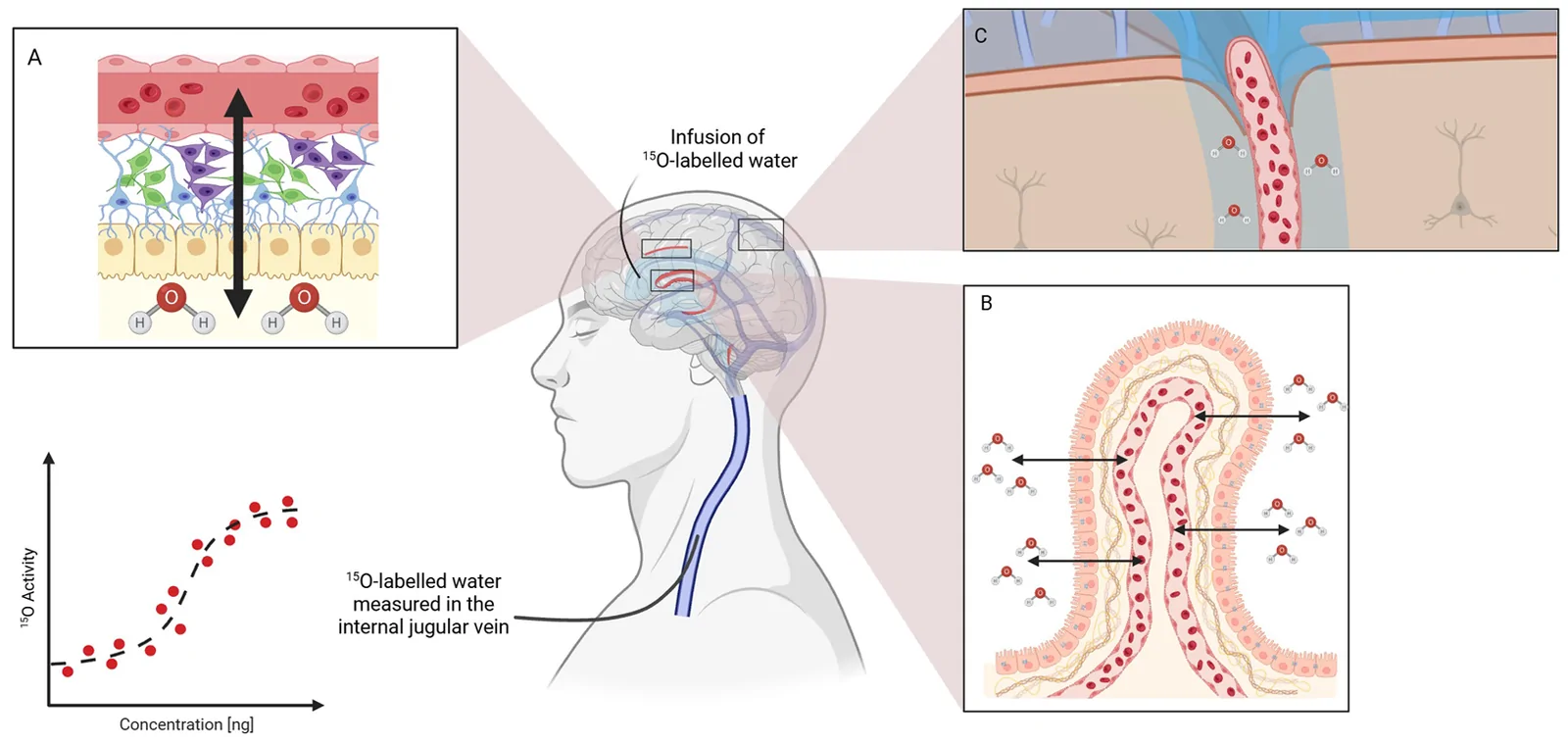
This is a visual representation of the abstract.

## Introduction

Water exchange between cerebrospinal fluid (CSF) and blood is a fundamental component of brain fluid homeostasis and reflects transport processes across the blood–CSF and blood–brain barriers. Previous studies have demonstrated that water moves rapidly and bidirectionally between CSF and the systemic circulation through distributed interfaces, including the choroid plexus, periventricular vasculature, pial vessels and perivascular pathways.^1^ Despite this conceptual framework, in vivo quantification of ventricular CSF-to-blood water transport kinetics has not been achieved in adult humans. As a result, the dynamics of ventricular water exchange remain incompletely characterized at the physiological level.

Classical isotope studies performed in the mid-20th century showed that heavy water introduced into the ventricles appears in the systemic circulation even in the presence of impaired CSF pathways, indicating persistent CSF–blood water exchange.^2,3^ However, these observations were not translated into a quantitative physiological parameter, nor examined in relation to intracranial pressure (ICP) dynamics, ventricular geometry or biochemical gradients. Consequently, ventricular CSF-to-blood water transport has remained largely descriptive rather than mechanistically defined in humans.

**Figure 1.**
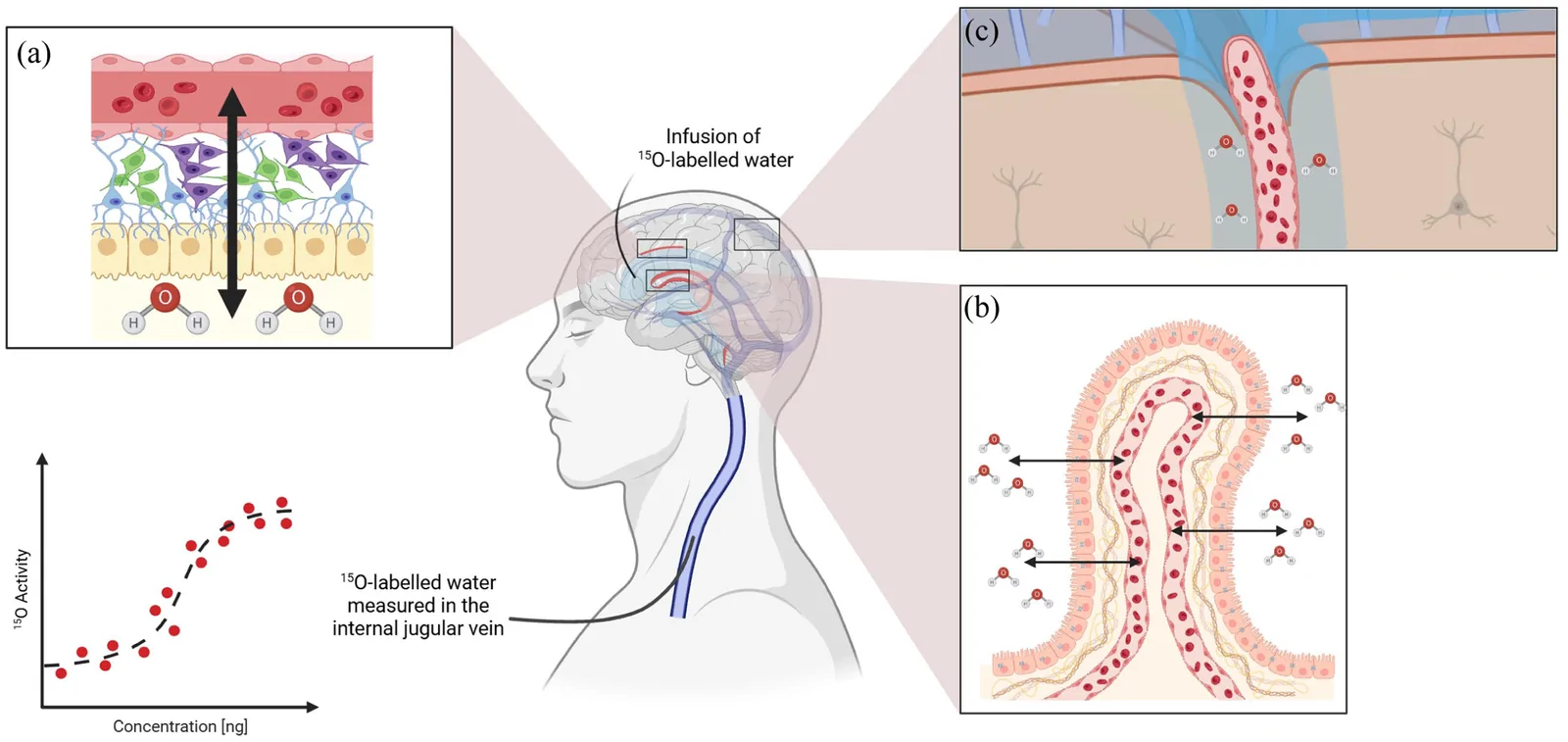
During ventricular infusion testing, ^15^O labelled water is infused to the lateral cerebral ventricle via an EVD and simultaneously venous blood is retrieved from the IJV. The passage route of ^15^O labelled water from ventricular CSF to venous blood of IJV is considered to be mainly via (a) the periventricular vasculature and probably also via (b) vasculature of the choroid plexus and to a lesser degree via (c) pial perivascular vessels. The measured activity of ^15^O labelled water in IJV is input to estimation of water absorption capacity using pharmacokinetic modelling (illustrated lower left). We consider the CSF–blood water exchange to be bidirectional, meaning that once ^15^O labelled has reached the vasculature from CSF, there also will be passage from blood to CSF. CSF: cerebrospinal fluid; EVD: external ventricular drainage; IJV: internal jugular vein.

In the present study, we introduce ventricular water absorption as a quantifiable physiological metric in adult hydrocephalus. Utilizing ventricular infusion testing, which has been clinical routine in this department for years,^4,5^ the present study combined intraventricular administration of ^15^O-labelled water with continuous jugular venous blood sampling and population pharmacokinetic modelling to estimate ventricular water absorption capacity. For illustration, see Figure 1. Moreover, we examined the relationship between ventricular water absorption and various other metrics including ICP dynamics, ventricular morphology and biochemical gradients between CSF and blood.

## Materials and methods

### Approvals and study design

The study was approved by the relevant regulatory and ethical authorities: the Regional Committee for Medical and Health Research Ethics (REK), Health Region South-East, Norway (455136); the Norwegian National Medicines Agency (EUDRACT 2022-002094-29); the Institutional Review Board at Oslo University Hospital (22/11415); and the Clinical Trial Information System (EU CT 2024-519933-53-00). The study was conducted in accordance with the Declaration of Helsinki and all participants provided written and oral informed consent. The study had a prospective, observational design.

### Study cohort

The study included adult patients referred to the Department of Neurosurgery, Oslo University Hospital – Rikshospitalet, Norway, for diagnostic evaluation of suspected adult hydrocephalus and assessment of the need for CSF diversion surgery. All participants had possible idiopathic normal pressure hydrocephalus (iNPH) in whom the indication for shunt surgery was uncertain. Based on age, history and imaging findings, there was a clinical indication for ventricular infusion testing in the included patients, in accordance with institutional routines. The clinical symptoms leading to diagnostic work-up were recorded.

As part of standard clinical practice, selected patients undergo overnight ICP monitoring, combined with ventricular infusion testing to inform surgical decision-making.^4^

### Surgical procedures

All procedures on day 1 were performed in the operating room under local anaesthesia and included retrograde catheterization of the right internal jugular vein and placement of a ventricular catheter with an ICP sensor.

#### Jugular venous catheterization

Retrograde catheterization of the right internal jugular vein (IJV) for jugular bulb blood sampling was performed with the patient in a slight Trendelenburg (head-down) position. The right IJV was punctured in its lower third under real-time ultrasound guidance using an 18-gauge introducer needle. After confirmation of venous blood return, a J-tipped guidewire was advanced cranially in a retrograde direction without resistance.

Correct intraluminal positioning of the guidewire was confirmed by ultrasound at the puncture site. The guidewire was advanced to the estimated depth corresponding to the jugular bulb, and a 7 Fr, 20 cm central venous catheter (ARROW^®^) was inserted over the guidewire using the Seldinger technique and advanced cranially toward the jugular bulb. The catheter tip was positioned at the estimated level of the jugular bulb and subsequently withdrawn approximately 1 cm to reduce the risk of vessel wall contact during sampling. Correct intravascular placement was confirmed by free venous backflow.

#### Ventricular catheter and ICP monitoring

A small right frontal skin incision was then made, followed by creation of a burr hole approximately 1 cm in diameter and a limited dural opening. An external ventricular drain (EVD) was inserted into the frontal horn of the lateral ventricle, with the distal catheter tip positioned at the level of the foramen of Monro. For ICP monitoring, either a Codman ICP MicroSensor (Codman & Shurtleff, Raynham, MA, USA) or a Raumedic ICP sensor (Raumedic AG, Helmbrechts, Germany) was used. The ICP sensor was placed in the brain parenchyma adjacent to the ventricular catheter. The burr hole was sealed with TachoSil^®^ to prevent cerebrospinal fluid leakage.

During the same operative session, CSF and venous blood samples were obtained for biochemical analyses, including measurement of osmolality and protein concentration.

### Overnight monitoring of static and pulsatile intracranial pressure

Following the surgical procedures on day 1, patients were transferred to the neuro-intermediate care unit for continuous overnight ICP monitoring. The continuous ICP signals were recorded using Sensometrics R&D Software (dPCom, Oslo, Norway), as previously described.^4,6^ The primary ICP metrics derived from overnight monitoring were:

- Mean ICP, representing the static component, and
- Mean ICP wave amplitude (MWA), representing the pulsatile component.

Continuous ICP monitoring was performed from days 1 to 2. On day 2, monitoring continued during ventricular infusion testing. All monitoring equipment was removed after completion of the infusion procedure, prior to patient discharge.

### Ventricular infusion testing and measurements of venous blood radioactivity

On day 2, patients were transferred to the Department of Nuclear Medicine for ventricular infusion testing. The test was performed with the patient awake. A bolus 10 mL of isotonic sodium chloride solution (NaCl 9 mg/mL; osmolarity 308 mOsm/L) containing ^15^O-labelled water was injected intraventricularly over 10 s via the external ventricular drain (EVD). Simultaneously, venous blood was sampled continuously from the jugular bulb catheter.

^15^O-labelled water was produced by the Norwegian Medical Cyclotron Centre in compliance with Good Manufacturing Practice (GMP). Owing to the short physical half-time of ^15^O (approximately 122 s), radiotracer production was performed near the infusion facility.

Blood radioactivity was recorded for 6 min at a sampling frequency of 2 Hz (flow rate 8 mL/min) using an automated blood sampling system (Allogg ABSS, Allogg AB, Mariefred, Sweden). Activity concentration was expressed in Bq/mL and corrected for detector dead time and radioactive decay to the time of injection. The administered activity was 650 MBq, corresponding to an estimated whole-body effective dose of approximately 0.7 mSv.^7^

Static and pulsatile ICP were recorded continuously throughout the infusion procedure. The principal ICP metrics during infusion were:

- The pressure–volume index (PVI),^8,9^ derived from mean ICP, and
- The maximum MWA observed during infusion.^4^

### Population pharmacokinetic modelling of ^15^O-labelled water passage from CSF to blood

Prior to population pharmacokinetic modelling, blood radioactivity data were pre-processed to reduce high-frequency noise inherent to radioactive measurements. Local polynomial regression smoothing was applied using a span of 0.25, selected based on visual inspection to preserve curve shape while reducing stochastic variation. Comparisons between raw and smoothed datasets are presented in Supplementary Figures 1 and 2.

Model selection was guided by goodness-of-fit criteria and physiological plausibility. A non-parametric population pharmacokinetic model was developed using the P_metrics_ package for R to characterize individual ventricular water absorption capacity. Jugular venous radioactivity–time profiles demonstrated a sigmoid-like shape.

The model employed an Erlang absorption model to capture the sigmoid-like shape of the pharmacokinetic profile of jugular venous radioactivity, using a total of four transit compartments prior to the central compartment, representing jugular venous blood. Alternative model structures were explored but did not improve fit (data not shown). All mass transfer used the same transport rate coefficient of absorption, *K_a_*. The mean transit time (MTT) for ^15^O-labelled water from site of infusion to jugular venous blood was calculated using MTT = 4/*K_a_* and from this the product of and the natural logarithm of two was calculated to obtain the cumulative half-time of the transit chain. This equates to the time taken for 50% available ^15^O-labelled water to pass through each transit compartment and is used as an index of ventricular water absorption capacity. This model-derived half-time (s) was used as the index of ventricular water absorption capacity and is hereafter referred to as ventricular water absorption. In this context, ventricular water absorption refers to the apparent clearance kinetics of intraventricular water under controlled infusion conditions and does not imply unidirectional bulk flow. An illustrative model used for modelling is shown in Supplementary Figure 1.

### Assessment of ventricular volume and surface area

Ventricular segmentation of the lateral, third and fourth ventricles was performed on T1-weighted magnetic resonance (MR) images using SynthSeg 2.0 in Freesurfer version 7.4.1.^10^ All automated segmentations were visually inspected and manually refined when necessary using PMOD version 4.3 (PMOD Technologies LLC, Zurich, Switzerland). Ventricular volumes and surface areas were extracted from the segmented regions of interest. Surface area was estimated by triangulation of the segmented ventricular contours in PMOD.

### Statistical analyses

Continuous variables are presented as mean ± standard deviation, and categorical variables as counts. Associations between variables were assessed using Pearson or Spearman correlation coefficients, as appropriate. Statistical significance was defined as a two-tailed *p* < 0.05. All statistical analyses were performed using IBM SPSS Statistics, version 31.

## Results

### Patient cohort

The study included 40 adult hydrocephalus patients who underwent overnight ICP monitoring followed by ventricular infusion testing with ^15^O-labelled water during the period 7 June 2023–3 December 2025. No patients were excluded; the patient characteristics are summarized in Table 1. The data refers to all 40 patients (except that two subjects lacked ICP scores during infusion testing). The clinical indication for diagnostic work-up reflected a combination of relatively young age for adult hydrocephalus (mean 60.5 ± 8.9 years), symptoms affecting quality of life and occupational capacity, and radiological ventriculomegaly (mean total ventricular volume 206.5 ± 115.0 mL). Examples of MR images showing ventricular morphology are shown in Supplementary Figure 2.

**Table 1.** Demographic and clinical characteristics, ventricular morphology, biochemical and intracranial pressure metrics of the study cohort examined for potential associations with ventricular water absorption.

Demographic	
Number	40
Age (years; Avg ± Stdev)	60.5 ± 8.9
Sex (F/M; *n*, %)	15 (37.5%)/25 (62.5%)
Weight (kg; Avg ± Stdev)	87.2 ± 19.4
Height (cm; Avg ± Stdev)	177.0 ± 10.5
Clinical symptoms	
Gait disturbance (*n*, %)	35 (88%)
Urinary urge/incontinence (*n*, %)	19 (48%)
Cognitive impairment (*n*, %)	33 (83%)
Headache (*n*, %)	15 (38%)
Nausea (*n*, %)	6 (15%)
Fatigue (*n*, %)	8 (20%)
Visual disturbances (*n*, %)	3 (8%)
Dizziness (*n*, %)	14 (35%)
Tinnitus (*n*, %)	2 (5%)
Duration (years; Avg ± Stdev)	2.4 ± 1.9
Ventricular volume	
Total (mL; Avg ± Stdev)	206.5 ± 115.0
Lateral ventricles (mL; Avg ± Stdev)	197.4 ± 112.4
Third ventricle (mL; Avg ± Stdev)	5.0 ± 2.6
Fourth ventricle (mL; Avg ± Stdev)	4.1 ± 1.7
Ventricular surface area	
Total (cm^2^; Avg ± Stdev)	379.7 ± 113.2
Lateral ventricles (cm^2^; Avg ± Stdev)	336.5 ± 107.1
Third ventricle (cm^2^; Avg ± Stdev)	19.6 ± 6.1
Fourth ventricle (cm^2^; Avg ± Stdev)	23.6 ± 4.1
Biochemical analyses	
Blood osmolality (mmol/kg; Avg ± Stdev)	287.5 ± 5.3
Blood protein (g/L; Avg ± Stdev)	66.3 ± 5.6
CSF osmolality (mmol/kg; Avg ± Stdev)	290.8 ± 4.3
CSF protein (g/L; Avg ± Stdev)	0.19 ± 0.09
Difference in osmolality (CSF–blood; mmol/kg; Avg ± Stdev)	3.6 ± 5.6
Difference in protein concentration (CSF–blood; g/L; Avg ± Stdev)	−65.5 ± 5.3
Overnight ICP score	
Mean ICP (mmHg; Avg ± Stdev)	7.5 ± 3.8
Mean ICP wave amplitude (mmHg; Avg ± Stdev)	4.6 ± 1.1
ICP during ventricular infusion test	
PVI (Avg ± Stdev)	23.0 ± 8.6
Maximum mean ICP wave amplitude <10, 10–15, >15 (*n*, %)	8 (21.1%), 11 (28.9%), 19 (50%)
Ventricular water absorption	
Half-time of absorption (s; Avg ± Stdev)	65.2 ± 34.3

Avg: average; ICP: intracranial pressure; PVI: pressure–volume index; Stdev: standard deviation.

### Overnight ICP monitoring

Overnight ICP metrics are presented in Table 1. Mean ICP was classified as abnormal (>15 mmHg) in 2/40 patients (5%), borderline (10–15 mmHg) in 9/40 (22.5%) and normal (<10 mmHg) in 29/40 (72.5%). Based on our established criteria,^6^ MWA was abnormal (>5 mmHg) in 17/40 patients (42.5%), borderline (4–5 mmHg) in 10/40 (25%) and normal (<4 mmHg) in 13/40 patients (32.5%).

### ICP during ventricular infusion testing

ICP was successfully recorded during ventricular infusion testing in 38/40 patients; in two patients the ICP sensor was inadvertently displaced prior to infusion. The mean pressure–volume index (PVI) was 23.0 ± 8.6 (Table 1). Based on previous work,^8,11^ PVI was categorized as borderline (10–15) in 6/38 patients (15.8%), mildly borderline (15–18) in 4/38 (10.5%) and normal (>18) in 28/38 patients (73.7%); no patient exhibited a PVI <10.

As no established reference ranges exist for pulsatile ICP during infusion, maximum MWA during infusion was categorized empirically as <10 mmHg in 8/38 patients (21.1%), 10–15 mmHg in 11/38 (28.9%) and >15 mmHg in 19/38 patients (50%).

### Safety

No procedure-related complications were observed. Ventricular infusion testing was well tolerated. In connection with the short-lasting infusion of 10 s, two patients (5%) reported transient headache that lasted less than 15 min; both patients also had comparable headache as part of their symptomatology prior to the infusion procedure. Another two patients reported transient frontal pressure without pain lasting less than a minute. The remaining 36/40 patients (90%) reported no discomfort or adverse sensations.

### Estimation of ventricular water absorption

**Figure 2.**
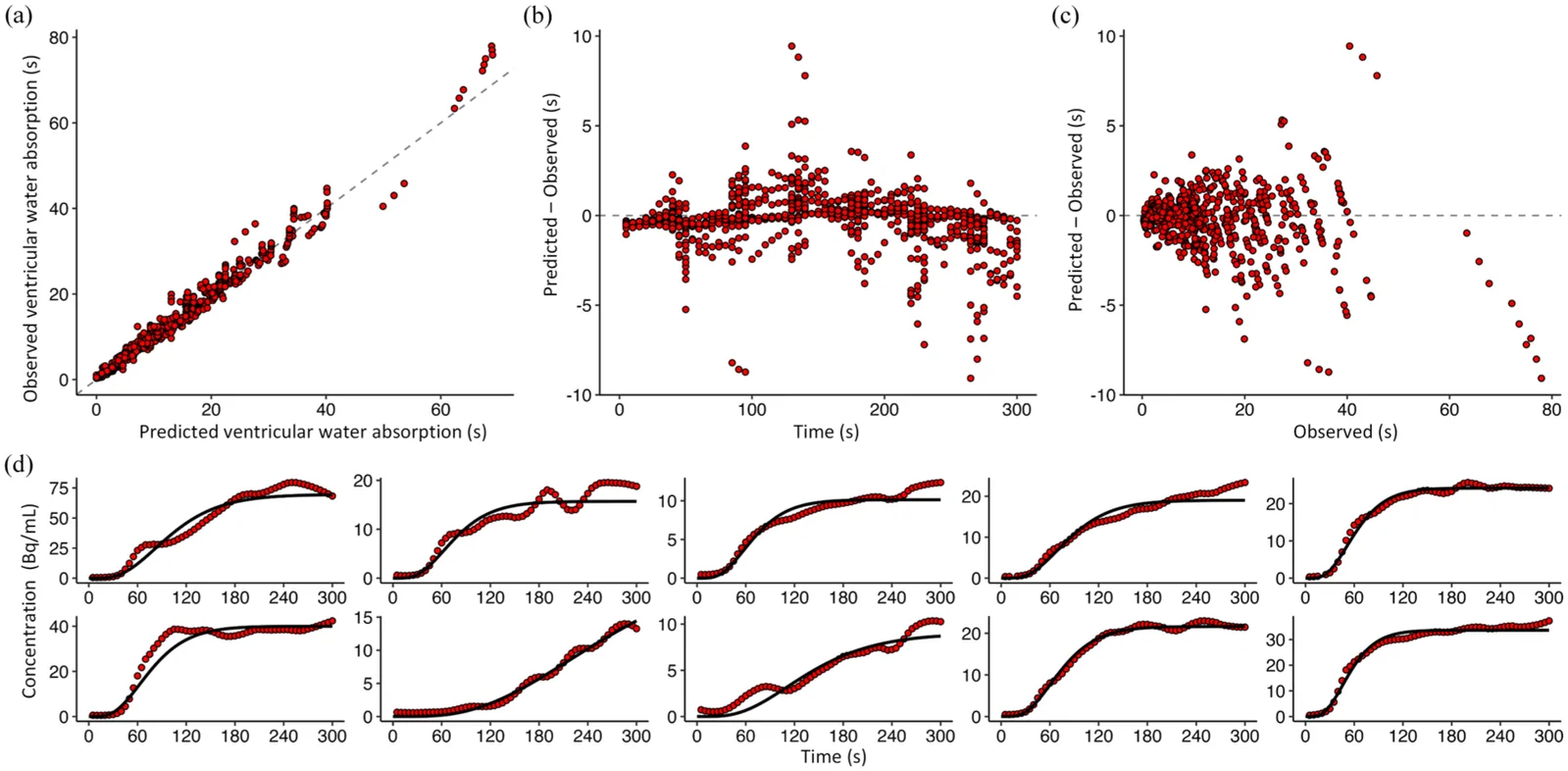
Estimation of ventricular water absorption using population pharmacokinetic modelling. Utilizing intraventricular administration of ^15^O-labelled water, ventricular water absorption was quantified using population pharmacokinetic methods, reflecting clearance of intraventricular water to the systemic circulation. Predictive performance is shown using (a) an observed versus predicted plot, (b) residuals as a function of time and (c) residuals as a function of observed concentration. The dashed line in a-c represents the unity. Together, these observations demonstrate satisfactory predictive performance. In (d), 10 random individual profiles of observed (red point) and model-predicted (black line) jugular venous blood radioactivity concentration. Due to very rich data, only every third datapoint is shown to improve readability and interpretation – the full version of this figure is shown in Supplementary Figure 1.

**Figure 3.**
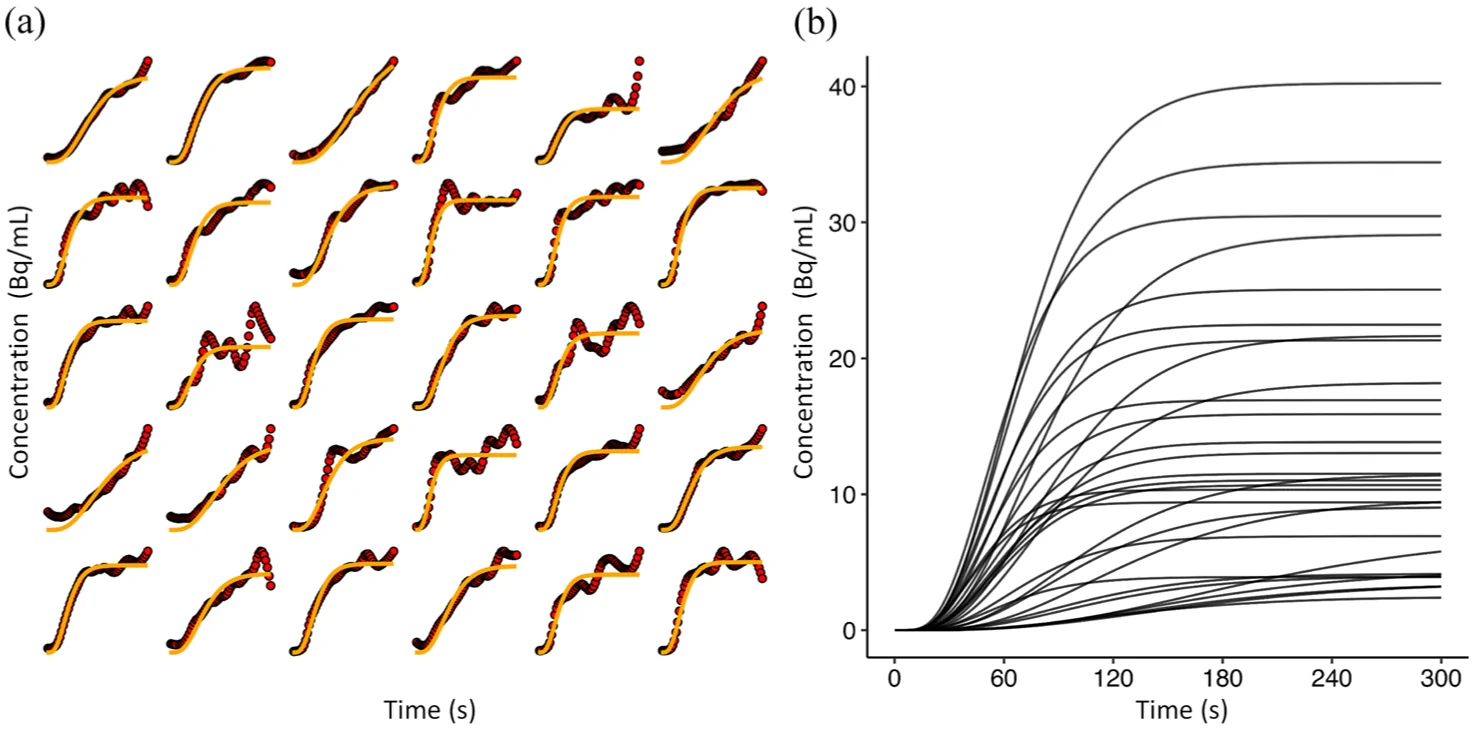
Inter-individual variability in pharmacokinetic profiles of ventricular water absorption. Visual representation of individual variability in ventricular water absorption, here demonstrated as (a) individual observed (red point) and model predicted (orange line) radioactivity concentration of ^15^O-labelled water and (b) individual model predicted concentration. This figure includes all subjects not shown in Figure 2(d).

Population pharmacokinetic modelling enabled estimation of ventricular water absorption in all included patients. Model predictions demonstrate satisfactory agreement between observed and predicted jugular venous radioactivity (Figure 2). Marked inter-individual variability was observed in the pharmacokinetics of jugular venous radioactivity profiles (Figure 3).

**Figure 4.**
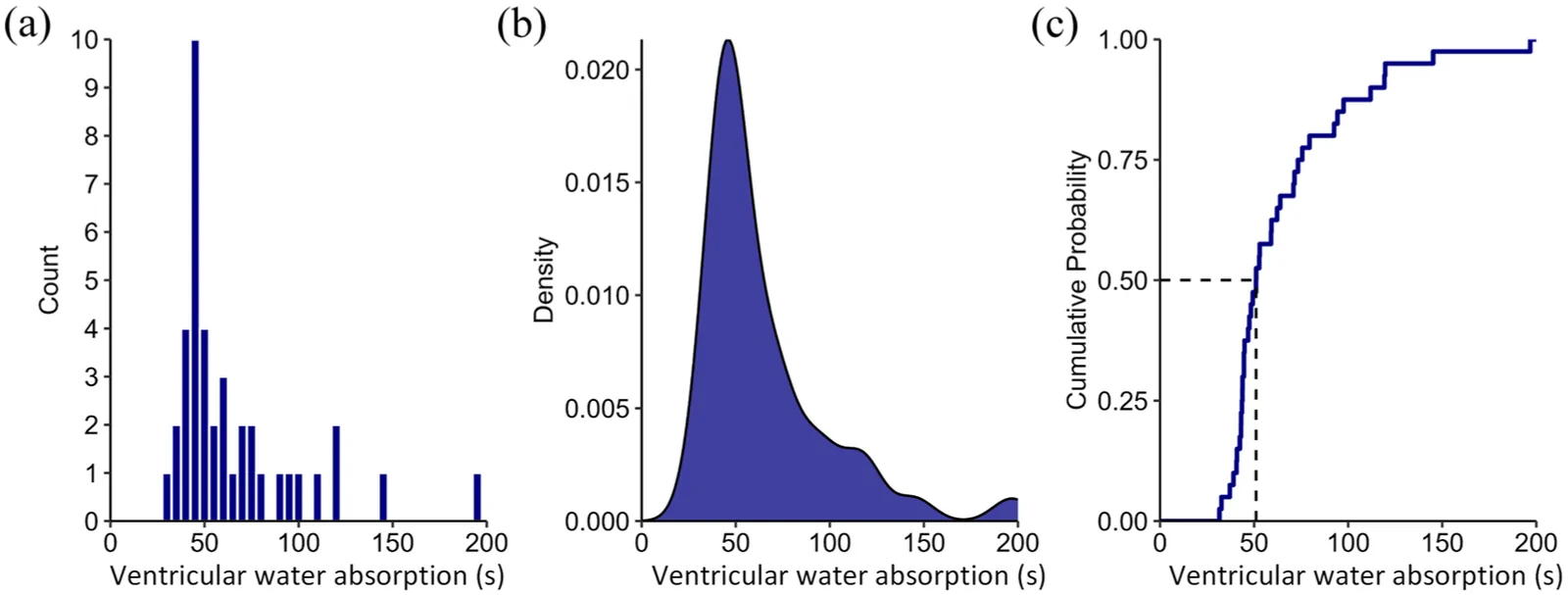
Distribution of ventricular water absorptive capacity. Estimates of ventricular water absorption (i.e. absorption half-time) from the population pharmacokinetic model. Figure demonstrates the (a) histogram (binwidth = 5), (b) kernel density estimate (bandwidth = 10) and (c) cumulative distribution function, where dotted lines indicate the median value of 51.06 s, close to the mode.

As no reference values are available, we thoroughly present the distribution of ventricular water absorption capacity in Figure 4. In this cohort, the mean ventricular water absorption (i.e. half-time of absorption) was 65.2 ± 34.3 s, while the median and interquartile range was 51.1 (43.7–73.9).

### Ventricular water absorption in relation to demographic and clinical variables

Ventricular water absorption was not associated with age (Pearson correlation (*R*) = −0.16, *p* = 0.312), height (*R* = 0.06, *p* = 0.734) or weight (*R* = −0.143, *p* = 0.378). No difference was observed between sexes (female: 62.2 ± 27.2 s, *n* = 15; male: 67.0 ± 38.3 s, *n* = 25). Similarly, ventricular water absorption did not differ according to the presence or absence of individual clinical symptoms (Supplementary Table 1).

### Ventricular water absorption in relation to intracranial pressure

**Figure 5.**
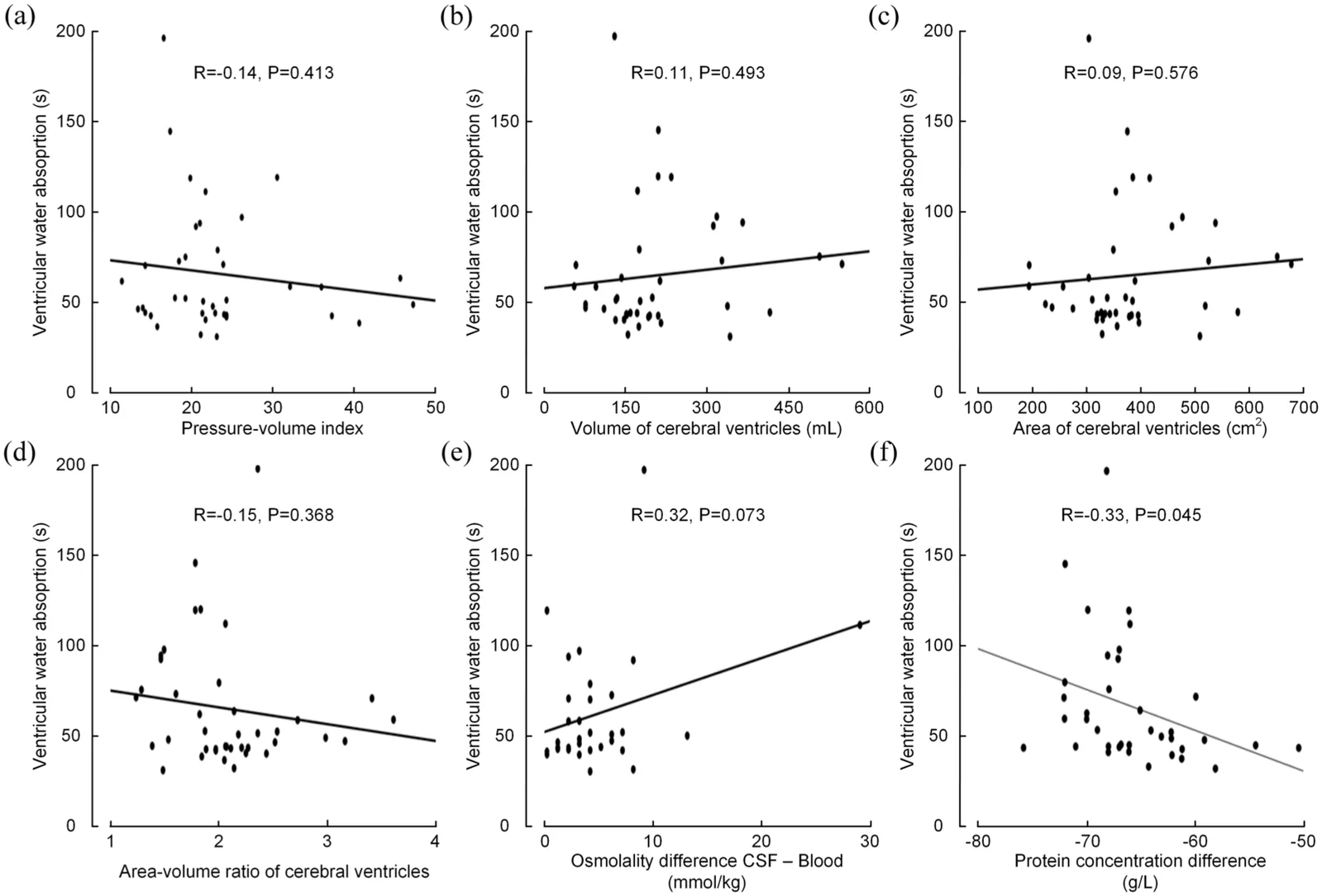
Ventricular water absorption is independent of intracranial pressure and ventricular geometry. Lack of association between ventricular water absorption and intracranial pressure, ventricular geometry and biochemical gradients. Ventricular water absorption did not correlate with (a) pressure–volume index, (b) total ventricular volume, (c) ventricular surface area, (d) ventricular surface-area-to-volume ratio, (e) CSF–blood osmolality difference, while (f) the correlation with CSF–blood protein concentration difference was signficant. These findings indicate that ventricular water absorption is not governed by global intracranial pressure, ventricular size, while modestly with bulk osmotic forces in adult hydrocephalus.

No association was observed between ventricular water absorption and ICP metrics obtained during overnight monitoring or ventricular infusion testing. Ventricular water absorption did not correlate with PVI (Figure 5(a)), mean overnight ICP (*R* = −0.11, *p* = 0.512) or overnight MWA (*R* = −0.04, *p* = 0.832; data not shown). Furthermore, ventricular water absorption did not differ across categories of normal, borderline or abnormal static or pulsatile ICP, either overnight or during infusion (Table 2).

**Table 2.** Ventricular water absorption for different categories of static and pulsatile ICP.

Static and pulsatile ICP scores	Ventricular water absorption (s)	Significance
Overnight ICP measures		
Mean ICP (mmHg) <10 mmHg (*normal*)	69.4 ± 38.1	*p* = 0.36
Mean ICP (mmHg) 10–15 mmHg (*borderline*)	58.0 ± 18.2	
Mean ICP (mmHg) ⩾15 mmHg (*abnormal*)	37.7 ± 8.7	
Mean ICP wave amplitude (mmHg) <4 mmHg (*normal*)	70.6 ± 31.4	*p* = 0.45
Mean ICP wave amplitude (mmHg) 4–5 mmHg (*borderline*)	53.3 ± 12.2	
Mean ICP wave amplitude (mmHg) ⩾5 mmHg (*abnormal*)	68.1 ± 43.8	
ICP measures during ventricular infusion testing		
Pressure–volume index ⩾18 (*normal*)	63.4 ± 26.1	*p* = 0.39
Pressure–volume index 10–18 (*borderline*)	74.7 ± 53.1	
Pressure–volume index <10 (*abnormal*)	–	
Maximum mean ICP wave amplitude (mmHg) <10 mmHg (*normal*)	64.3 ± 30.0	*p* = 0.70
Maximum mean ICP wave amplitude (mmHg) 10–15 mmHg (*borderline*)	60.0 ± 20.0	
Maximum mean ICP wave amplitude (mmHg) ⩾15 mmHg (*abnormal*)	71.0 ± 43.3	

ANOVA: analysis of variance.

Statistical differences between groups were determined by ANOVA with Bonferroni corrected post hoc tests. There were neither any significant differences between the sub-groups.

### Ventricular water absorption in relation to ventricular morphology

Ventricular water absorption was not correlated with total ventricular volume (Figure 5(b)), ventricular surface area (Figure 5(c)) or surface-area-to-volume ratio (Figure 5(d)). Analyses performed separately for the lateral, third and fourth ventricles likewise revealed no associations between ventricular water absorption and regional ventricular volume, surface area or surface-area-to-volume ratios (data not shown).

### Ventricular water absorption in relation to biochemical variables

Ventricular water absorption did not correlate with osmolality in CSF (*R* = 0.18, *p* = 0.275), blood (*R* = −0.06, *p* = 0.738) or the CSF–blood osmolality difference (Figure 5(e)). No significant associations were observed with protein concentration in CSF (*R* = −0.08, *p* = 0.654) or blood (*R* = 0.27, *p* = 0.099). However, ventricular water absorption is modestly associated with the difference in protein concentration between the CSF and blood (Figure 5(f)), indicating higher absorption with relatively greater protein concentration in blood than in CSF.

## Discussion

The present study provides, to our knowledge, the first quantitative in vivo estimates of ventricular CSF-to-blood water transport kinetics in adult humans. By combining intraventricular administration of ^15^O-labelled water with continuous jugular venous sampling, high-temporal-resolution ICP monitoring and non-parametric population pharmacokinetic modelling, we demonstrate that ventricular water exchange can be characterized as a measurable kinetic parameter at the individual level. Ventricular water transport exhibited substantial inter-individual variability, with an average absorption half-time of approximately 1 min.

Although jugular bulb sampling preferentially reflects cerebral venous outflow, contributions from extracerebral tissues and vascular mixing cannot be entirely excluded. However, given the intraventricular administration and the use of a population pharmacokinetic model over a short time scale, the estimated rate of absorption is expected to be dominated by cerebral exchange of CSF and blood.

The estimated transport half-times in the present study (~30–200 s) are consistent with prior experimental and imaging studies of CSF–blood water exchange.^1^ Tracer studies have demonstrated rapid appearance of water in venous blood within seconds to minutes, with reported time constants of approximately 14 s for pial pathways and ~60–80 s for blood–CSF exchange assessed by MRI techniques.^1^ These observations support the interpretation that ventricular CSF-to-blood water transport occurs on a timescale of seconds to minutes and is consistent with a diffusion-dominated process, with potential modulation by hydrostatic and vascular forces.

Current knowledge of cerebral water homeostasis remains limited and is based on a relatively small and heterogeneous body of experimental and clinical studies. A recent systematic review identified only 18 studies over more than 70 years that directly addressed water exchange across blood–CSF barriers.^1^ Collectively, this literature supports the concept that water moves freely, rapidly and bidirectionally between CSF and blood across multiple interfaces along the craniospinal axis, including the choroid plexus, ventricular ependyma, periventricular capillaries, pial vessels and perivascular spaces. Importantly, net water movement at any site reflects the balance of local hydrostatic, osmotic and molecular gradients, with diffusion as the dominant transport mechanism and modulation by aquaporins and vascular forces.^1^ In this framework, CSF water exchange should be regarded as a distributed and continuous process rather than a unidirectional flow between defined production and absorption sites. However, despite this conceptual framework, ventricular water absorption has not previously been defined or quantified as a physiological parameter in adult humans or in adult hydrocephalus.

The only direct human data on ventricular CSF-to-blood water passage derive from early heavy-water (D_2_O) studies performed in the mid-20th century. In hydrocephalic infants, Bering^2,3^ reported markedly prolonged half-times for D_2_O exchange between ventricular CSF and blood and attributed this to a reduced ventricular surface-area-to-volume ratio. These seminal observations established that ventricular water exchange persists even in the presence of obstructed CSF pathways, but they were not developed into a quantitative metric applicable to adult hydrocephalus, nor were they examined in relation to ICP dynamics or biochemical gradients. In contrast, the present study introduces ventricular water absorption as a model-derived physiological parameter, estimated at the individual level in adult patients and validated against measured concentration–time data. Notably, the present results disclosed no association between ventricular water absorption and the ventricular surface area-volume ratio, suggesting that geometric factors alone do not determine exchange kinetics within the physiological range studied.

Water absorption was estimated during bolus ventricular infusion testing, which is associated with transient elevation of intraventricular pressure, which is expected to influence water movement. Experimental studies have demonstrated that increased intraventricular pressure enhances efflux of tritiated water from ventricles to blood,^12^ indicating a pressure-dependent component superimposed on diffusion, while increased choroid plexus perfusion or arterial pressure augments blood-to-CSF water influx.^13^ Against this background, the absence of any association between ventricular water absorption and static or pulsatile intracranial pressure, whether assessed overnight or during infusion, was unexpected. This lack of association was consistent across multiple pressure metrics, including the pressure–volume index and mean ICP wave amplitude, suggesting that ventricular water absorption is not strongly associated with global ICP or compliance within the ranges observed in this cohort. Rather, these findings support the interpretation that water exchange is dominated by the intrinsic permeability of the CSF–blood interfaces rather than by pressure-driven bulk flow.

From this study, we cannot precisely define the anatomical site of water exchange. We consider the periventricular / subependymal vasculature to be a major contributor (Figure 1(a)), although the choroid plexus vasculature (Figure 1(b)) and pial vessels within the subarachnoid space (Figure 1(c)) are also likely involved. Given the short time scale and the fact that the injected ^15^O labelled water initially resides within the ventricular system, we consider the term ventricular water absorption justified as an operational definition. However, CSF–blood water exchange is inherently bidirectional. Over the observed time scale (mean ~65 s), blood circulates through the brain multiple times, and labelled water may repeatedly cross the CSF–blood interface. Accordingly, the measured signal reflects net exchange kinetics within a rapidly equilibrating system rather than a strictly unidirectional absorption process. Given the short observation window, the measured kinetics likely emphasize early exchange processes, including periventricular and vascular interfaces, and should therefore be interpreted as an integrated measure of ventricular-region water exchange rather than a strictly ventricular surface phenomenon.

A modest negative association was observed between the CSF–blood protein concentration difference and ventricular water absorption, consistent with the possibility that oncotic pressure gradients contribute to water movement from CSF to blood. However, ventricular water absorption did not correlate with CSF or blood osmolality, nor with the CSF–blood osmotic difference. This may appear counterintuitive, as experimental manipulation of osmotic gradients – such as intrathecal administration of hyperosmolar mannitol – can reverse the direction of CSF–blood water flux.^14^ The present findings suggest that, under the conditions studied in adult hydrocephalus, bulk osmotic forces are not the primary determinant of ventricular water absorption capacity, and that diffusion-driven exchange dominates within the physiological range.

Given the cohort size (*n* = 40), the study may be underpowered to detect small-to-moderate associations, and subtle effects cannot be excluded. Accordingly, the absence of significant correlations should be interpreted with caution, particularly for variables with limited physiological range within this population.

Collectively, these findings indicate that ventricular water absorption is regulated independently of ventricular size, ICP and bulk osmotic forces. One plausible explanation is that water absorption reflects intrinsic properties of the brain’s microvascular and perivascular exchange systems. In this context, our systematic review^1^ highlighted the central role of aquaporin-4 (AQP4) in facilitating water movement across brain fluid compartments. In vivo imaging and genetic models^15,16^ demonstrate that deletion or loss of AQP4 polarization markedly reduces fluid and solute flux between perivascular and interstitial compartments, while pharmacological facilitation of AQP4 (e.g. TGN-073) enhances cortical water flux and increases interstitial fluid turnover.^17^ Although AQP4 function was not assessed directly in the present study, such mechanisms may contribute to the observed inter-individual variability in ventricular water absorption and further support a permeability-driven model of water exchange.

The pharmacokinetic model represents a simplified compartmental approximation of a distributed exchange system, and individual parameters should therefore be interpreted as effective transport kinetics rather than direct anatomical or molecular rate constants.

Ventricular water absorption is likely to be particularly relevant in adult hydrocephalus, where reflux of CSF into the lateral and third ventricles is a consistent imaging feature. Phase-contrast MRI^18,19^ has demonstrated ventricular reflux in iNPH, with higher reflux grades associated with abnormal pulsatile ICP.^18,20^ and favourable response to shunt surgery. These observations suggest that the ventricles are not passive reservoirs but active sites of fluid exchange, reinforcing the rationale for quantifying ventricular water absorption directly.

Beyond hydrocephalus, altered water exchange across blood–CSF barriers may have broader relevance for ageing, vascular diseases and neurodegeneration. Ventricular and subarachnoid space volumes increase with age,^21,22^ and are further enlarged in conditions such as Alzheimer’s disease^23^ and arterial hypertension.^24,25^ Experimental imaging studies have demonstrated reduced blood–CSF water exchange in both arterial hypertension^26^ and transgenic models of Alzheimer’s disease.^27^ This raises the possibility that impaired water handling contributes to altered fluid homeostasis and clearance mechanisms. Whether ventricular water absorption capacity changes across the lifespan or in other neurological disorders remains an important question for future investigation.

An important consideration is whether the efflux routes for the solvent (water) and solute components of CSF are the same. While there is increasing interest in human CSF-mediated solute clearance pathways,^28^ including glymphatic transport,^29^ these mechanisms primarily describe perivascular and interstitial movement of solutes and should not be conflated with water exchange. CSF consists predominantly of water (99%), and the kinetics of water exchange differ markedly from those of solutes. As demonstrated here, water exchange occurs on a time scale of seconds (mean 65.2 ± 34.3 s, ranges 31.5–196.8 s), whereas clearance of solutes such as intrathecal tracers occurs over hours. For example, among individuals with iNPH, brain fluid clearance to blood of an intrathecal tracer (gadobutrol) with molecular weight 604 Da (expressed as half-time of absorption (*T*_1/2, abs_)) was 4.2 ± 3.1 h.^30^ This distinction underscores that solvent and solute components of CSF follow partially separate pathways and operate on different spatial and temporal scales. In this context, CSF–blood water exchange may be regarded as a microscale process of continuous renewal, whereas glymphatic transport reflects macroscale convective movement of solutes. The clearance pathways of solutes in humans (e.g. tracers administered to CSF) have not been entirely determined but seem to occur via the meninges (and meningeal lymphatics),^31^ cranial nerves,^32^ choroid plexus,^33^ and to some extent across BBB,^34^ while nasal lymphatics may play a less role in humans.^35^ Several limitations of this study should be acknowledged. First, ventricular water absorption was estimated during ventricular infusion testing, a condition that does not represent physiological steady state, as intraventricular volume and pressure are transiently increased. On the other hand, all patients were studied under identical perturbation conditions. The inter-individual variability most likely reflects intrinsic differences. Notably, this controlled perturbation of ventricular infusion enabled reproducible tracer delivery and simultaneous assessment of intracranial pressure dynamics. The short physical half-time of ^15^O-labelled water limits prolonged non-physiological exposure. Moreover, pressure-dependent effects on water exchange would be expected to strengthen, rather than obscure, associations between absorption capacity and ICP, yet no such relationships were observed.

Second, the invasive nature of ventricular infusion testing may be considered a limitation. For decades, this department^4,5^ and others^36,37^ have used ventricular infusion testing in work-up of hydrocephalus patients. The procedure was clinically indicated in all participants, was well tolerated, and was not associated with adverse events in this cohort. The only complaint during infusion was short-lasting headache during the infusion in 2/40 (5%) subjects, while another two individuals report short-lasting pressure, but not pain, in the head during infusion. The others reported no complaints. Importantly, the combination of direct ventricular access, continuous jugular venous sampling and high-temporal-resolution pressure monitoring provided a level of physiological characterization, that is, not achievable with non-invasive techniques. Ethical considerations preclude application of this approach in healthy individuals, limiting the availability of normative data. However, the method is most relevant in patients with suspected hydrocephalus, where improved characterization of brain water handling may have clinical relevance.

Third, the short half-time of ^15^O-labelled water imposes logistical constraints and limits widespread clinical implementation. While this restricts routine use, it does not detract from the conceptual significance of demonstrating that ventricular water absorption can be quantified in vivo. Future methodological developments may enable less invasive or more widely accessible approaches to assess water exchange across CSF–blood barriers.

Fourth, the absence of an association between ventricular water absorption and intracranial pressure metrics might be attributed to the fact that ICP was measured in the brain parenchyma rather than directly within the ventricular system. Small pressure gradients between ventricular CSF and parenchyma have been reported during acute stages of hydrocephalus,^38^ although such gradients are minimal in adult hydrocephalus.^39^ Moreover, from our previous studies, pressures measured in ventricular CSF and brain parenchyma correlate closely. Therefore, from our prior experience, it seems unlikely that ventricular–parenchymal pressure gradients account for the lack of association between ventricular water absorption and ICP observed in the present study.

Finally, due to the burden of the investigation we have only performed a single measurement in each individual. As such, we are unable to estimate the intraindividual variability in ventricular water absorption. Future studies should assess test–retest reproducibility to determine whether ventricular water absorption represents a stable individual trait.

## Conclusions

In conclusion, the present study demonstrates that ventricular CSF-to-blood water transport can be quantified and constitutes a distinct physiological metric with substantial inter-individual variability in adults with hydrocephalus. Using intraventricular administration of ^15^O-labelled water combined with population pharmacokinetic modelling, we derived individual estimates of ventricular water absorption kinetics, which demonstrated close agreement between measured and model-predicted blood activity. Contrary to prevailing assumptions, ventricular water transport was not associated with ICP, whether assessed as static or pulsatile components, nor with ventricular volume, ventricular surface area or osmotic gradients between CSF and blood within the physiological range studied. These findings suggest that ventricular water exchange is governed primarily by intrinsic properties of CSF–blood interfaces, consistent with a diffusion-dominated and distributed exchange rather than pressure-driven bulk flow. By introducing ventricular water absorption as a measurable physiological parameter, this work extends previous tracer-based observations and contributes to a more physiologically grounded understanding of brain fluid homeostasis. The methodology and findings establish a framework for future studies investigating how altered water exchange may contribute to hydrocephalus, ageing and neurological disease.

## Supplemental Material

sj-pdf-1-jcb-10.1177_0271678X261466792 – Supplemental material for Ventricular CSF-to-blood water transport kinetics in adult hydrocephalus Supplemental material, sj-pdf-1-jcb-10.1177_0271678X261466792 for Ventricular CSF-to-blood water transport kinetics in adult hydrocephalus by Per Kristian Eide, Alexander Gul Sherwani, Luis Romundstad, Jan Gunnar Fjeld, Amanda Eriksson, Trine Hjørnevik and Markus Hovd in Journal of Cerebral Blood Flow & Metabolism
