# Supplementary material for: Ventricular CSF-to-blood water transport kinetics in adult hydrocephalus
Source: J Cereb Blood Flow Metab. 2026 Jun 29:0271678X261466792. Online ahead of print. doi: 10.1177/0271678X261466792 (PMC13558470; doi:10.1177/0271678X261466792)
Supplement: Supplementary file 1 [file sj-pdf-1-jcb-10.1177_0271678X261466792.pdf]

Supplementary Material

Ventricular CSF-to-blood water transport kinetics in adult hydrocephalus

Per Kristian Eide,[1,2,3,*] Alexander Gul Sherwani,[4] Luis Romundstad,[5] Jan Gunnar Fjeld,[4] Amanda Eriksson,[2,3,6] Trine Hjørnevik,[2,3,6] Markus Hovd[7,8]

Author affiliations:
[1]Department of Neurosurgery, Oslo University Hospital – Rikshospitalet, Oslo, Norway.
[2]Institute of Clinical Medicine, Faculty of Medicine, University of Oslo, Oslo, Norway.
[3]K.G. Jebsen Centre for Brain Fluid Research, University of Oslo, Oslo, Norway.
[4]Department of Nuclear Medicine, Oslo University Hospital – Rikshospitalet, Oslo, Norway.
[5]Department of Anesthesia and Intensive Care medicine, Division of Emergencies and Critical care, Oslo University Hospital, Oslo, Norway.
[6]Department of Physics and Computational Radiology, Oslo University Hospital, Oslo, Norway.
[7]Department of Pharmacy, University of Oslo, Oslo, Norway.
[8]Department of Transplantation Medicine, Oslo University Hospital, Oslo, Norway.

Supplementary Figure 1

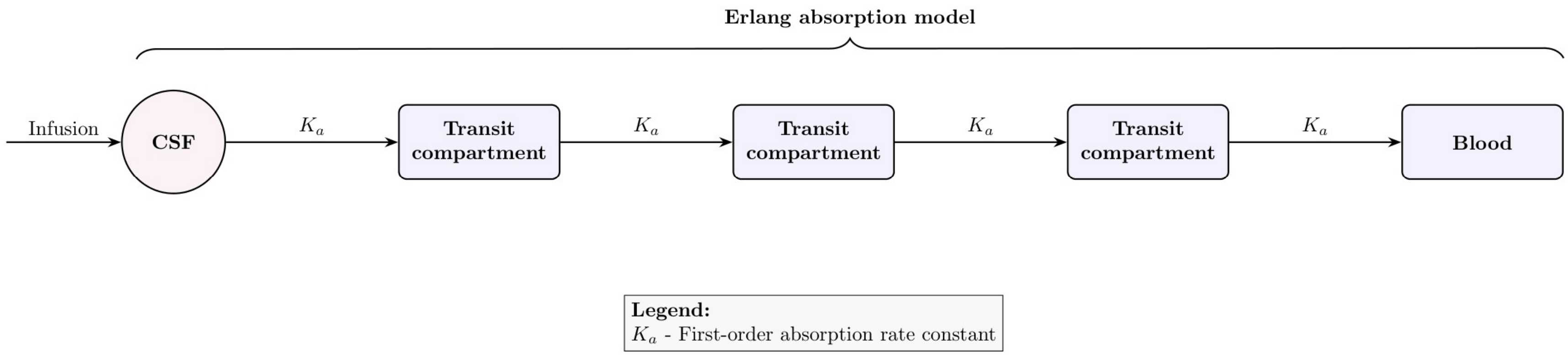

Structural overview of the population pharmacokinetic model, demonstrating absorption of ^{15}O labeled water from ventricular cerebrospinal fluid (CSF) following intraventricular injection. An Erlang absorption model parameterized by the first-order absorption rate coefficient Ka is assumed to mediate absorption from CSF to blood, from which measurements are taken.

Supplementary Figure 2

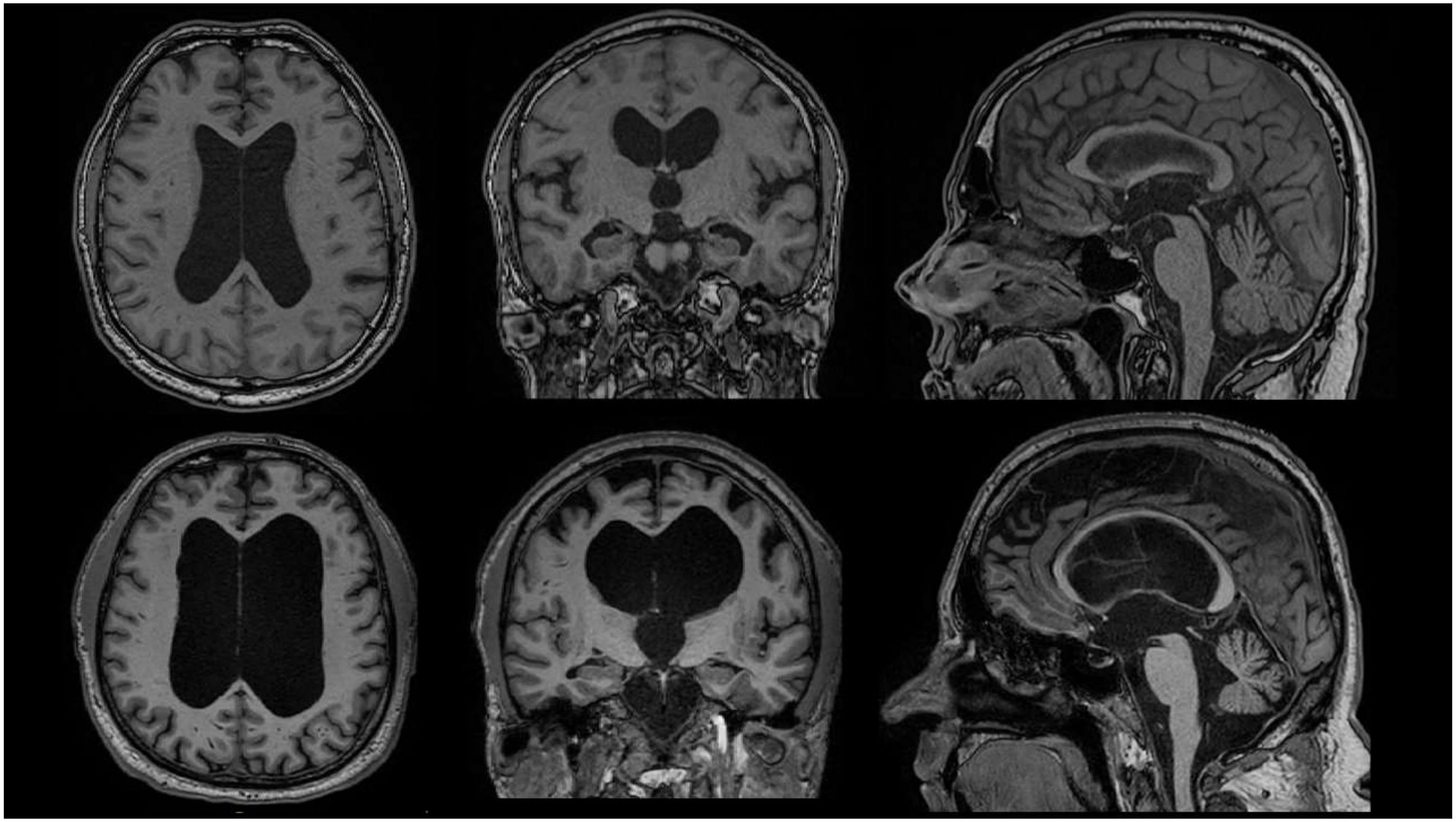

Two illustrative patients included in the study. The T1 images of one subject is shown in the upper row, and another subject in the lower row.

Supplementary Figure 3

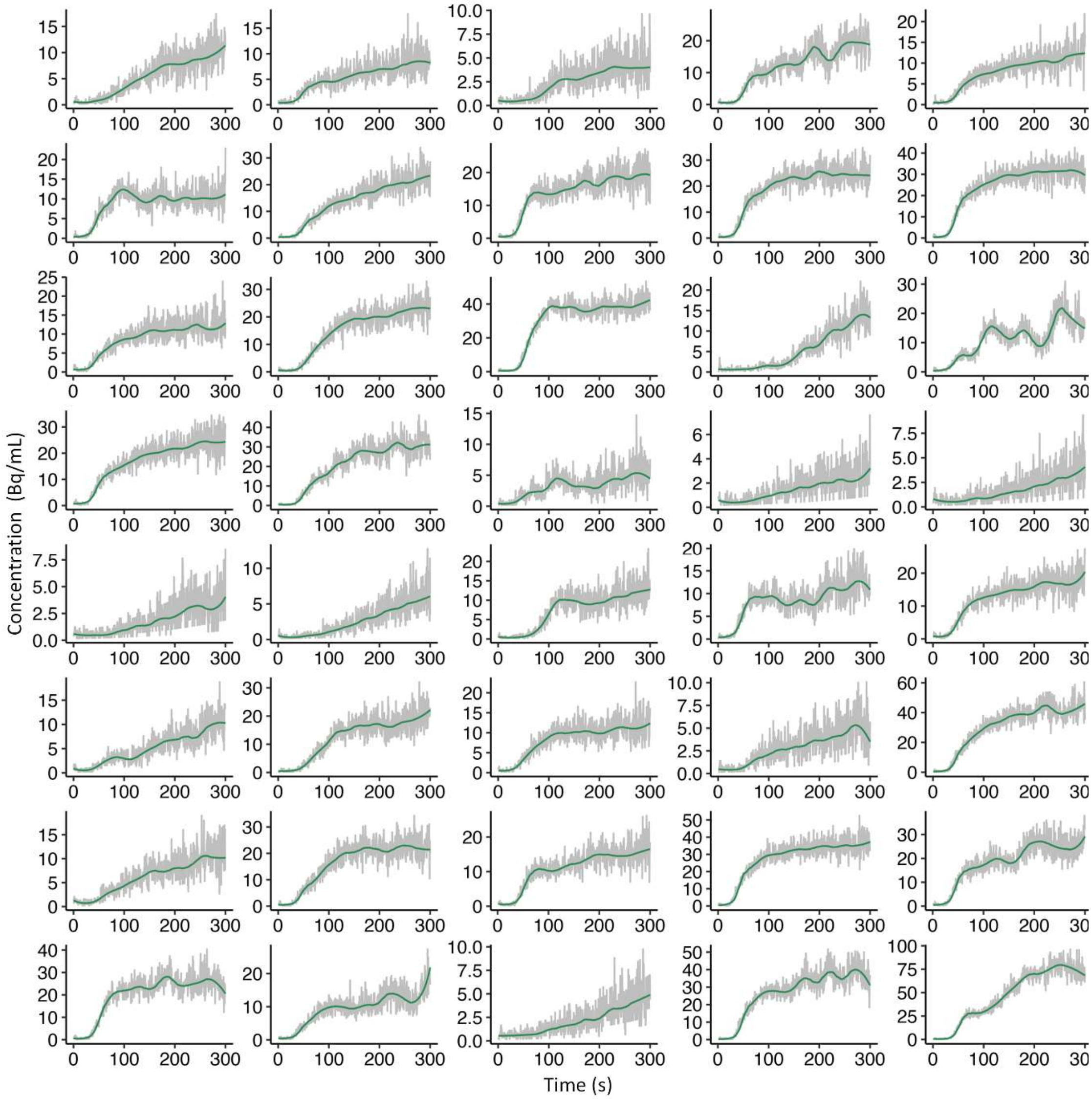

Raw and smooth jugular venous activity. For each individual, the noisy raw data is shown in light gray. The green line is the result of a local polynomial regression smoothing using a span of 0.25 and was used for population pharmacokinetic modelling to estimate individual ventricular water absorption.

Supplementary Figure 4

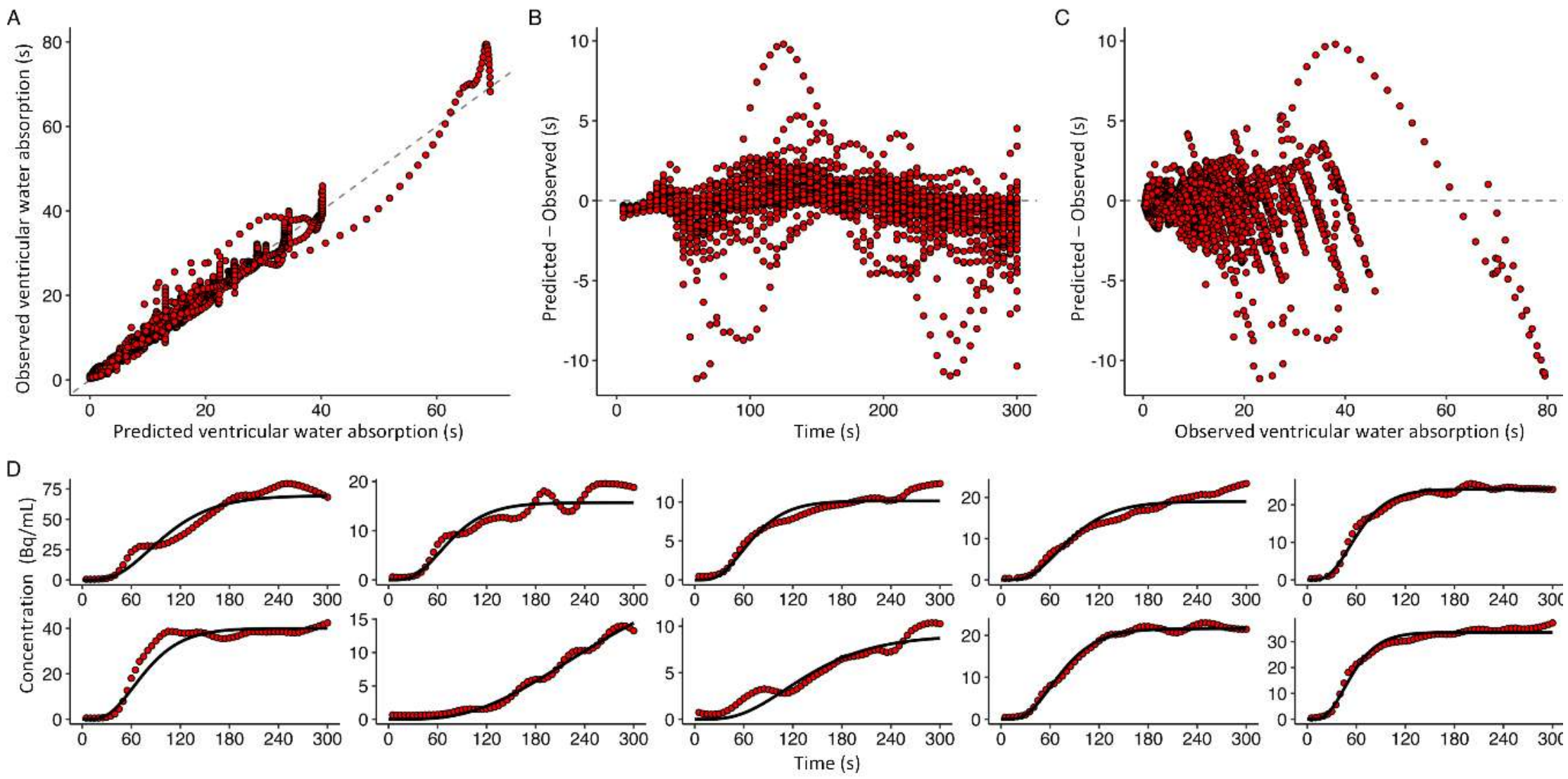

Full version of Figure 1. Predictive performance is shown using A) an observed versus predicted plot of ventricular water absorption, B) residuals as a function of time, and C) residuals as a function of observed concentration. Together, these demonstrate satisfactory predictive performance. In D), ten random individual profiles of observed (red point) and model-predicted (black line) jugular venous activity.

Supplementary Table 1
Ventricular water absorption for different symptoms leading to work-up

	Ventricular water absorption (s)	Significance
**Gait disturbance**		
Present (n=35)	62.5±28.2	P=0.186
Absent (n=5)	84.3±64.5	
**Urinary urge / incontinence**		
Present (n=19)	64.1±30.2	P=0.844
Absent (n=21)	66.2±38.4	
**Cognitive impairment**		
Present (n=33)	65.8±33.9	P=0.826
Absent (n=7)	62.6±38.5	
**Headache**		
Present (n=15)	65.4±39.8	P=0.977
Absent (n=25)	65.1±31.4	
**Nausea**		
Present (n=6)	68.2±63.3	P=0.818
Absent (n=34)	64.7±27.9	
**Fatigue**		
Present (n=8)	84.3±59.0	P=0.079
Absent (n=32)	60.4±24.0	
**Visual disturbances**		
Present (n=3)	45.4±15.6	P=0.304
Absent (n=37)	66.8±35.0	
**Dizziness**		
Present (n=14)	71.4±45.4	P=0.406
Absent (n=26)	61.9±27.0	
**Tinnitus**		
Present (n=2)	52.6±13.6	P=0.601
Absent (n=38)	65.9±35.0	

Statistical differences between groups were determined by analysis of variance with Bonferroni corrected post hoc-tests. There were neither any significant differences between the sub-groups.
